# Finding the Mole and Choosing the Apple: Executive Function Challenges in Children with Developmental Language Disorder (DLD)

**DOI:** 10.3390/children11121519

**Published:** 2024-12-14

**Authors:** Soraya Sanhueza, Mabel Urrutia, Hipólito Marrero

**Affiliations:** 1 Facultad de Humanidades y Arte, Universidad de Concepción, Concepción 4070386, Chile; sorayasanhueza@udec.cl; 2 Facultad de Educación, Universidad de Concepción, Concepción 4070386, Chile; 3 Facultad de Psicología y Logopedia, Instituto Universitario de Neurociencias de la Universidad de La Laguna (IUNE), Universidad de La Laguna, 38200 La Laguna, Spain; hmarrero@ull.edu.es

**Keywords:** developmental language disorder (DLD), sustained attention, working memory, executive functions, preschool children

## Abstract

Background: Children with a developmental language disorder (DLD) frequently experience deficits in cognitive skills such as working memory (WM) and sustained attention (SA), which are closely related to language development. Yet, these cognitive deficits remain underexplored in early childhood, particularly during the preschool years. Objective: This study explores WM and SA in Chilean preschoolers with a DLD compared to their typically developing (TD) peers, using the nonverbal tasks “Torpo the Clumsy Mole” for WM and the Continuous Performance Task (CPT) “Duno and the Worms” for SA, both from the Child Neuropsychological Evaluation Test (TENI in Spanish). Method: Thirty DLD and 30 TLD peers (aged 4 to 4 years 11 months) participated. Accuracy and reaction times in both tasks were assessed. Results: The children with a DLD demonstrated significant deficits in working memory accuracy and poorer sustained attention accuracy despite exhibiting shorter reaction times in the sustained attention task compared to TLD children. Conclusions: The findings highlight the multifaceted nature of a DLD, particularly in relation to cognitive dimensions beyond language, such as working memory and sustained attention. Early identification of these differences emphasizes the important role of executive functions in DLDs.

## 1. Introduction

Developmental language disorder (DLD) is a common condition observed during an individual’s school years, characterized by deficiencies in acquiring and utilizing morphosyntax, semantics, vocabulary, phonology, and complex syntax [1]. Since its initial diagnosis, research efforts have been undertaken to better delineate this population and understand the underlying causes of their language difficulties. Notably, these investigations have highlighted cognitive aspects that influenced the nomenclature of the diagnosis. Previously called Specific Language Impairment (SLI), this condition was viewed as limited to the linguistic domain. However, contemporary evidence suggests that non-linguistic areas, such as learning, attention, and memory, are also affected [2].

The cognitive and linguistic challenges associated with DLDs pose a significant barrier, especially during the early school years, a critical developmental phase when acquired skills can have lasting consequences [3]. Substantial evidence supports the critical role of cognitive abilities in language performance [4,5]. This underscores the importance of characterizing these cognitive abilities to enhance the prognosis for individuals with a DLD.

Among the various cognitive domains investigated, executive functions (EFs) have garnered particular attention. EFs encompass the cognitive abilities required for planning, organizing, and executing goal-oriented actions [6]. These functions exhibit a sequential developmental pattern, intensifying during childhood and plateauing in adolescence. However, their manifestation can vary significantly, especially among pediatric populations [7]. While some studies suggest that all EF components distinctly manifest at an early age, others propose that a single component better explains the variance in EF measures [8,9].

As a relevant component of EF, working memory has received considerable scrutiny. Its development has been observed during early childhood [10,11], playing a vital role in updating information while performing complex tasks [6,12]. Impairments in working memory in children with a DLD may lead to syntactic and semantic challenges, difficulties in speech planning, and disruptions in phonological development [13]. Working memory deficits among these children were confirmed both in verbal and in nonverbal tasks [14,15,16]. Furthermore, an association was established between nonverbal working memory and the severity of language impairment [17]. Although there is a consensus about the implication of working memory in linguistic performance, recent research suggests that sustained attention, another critical cognitive skill, may serve as a mediating factor in the relationship between nonverbal working memory and language skills in children with a DLD [18]. 

Sustained attention, which encompasses the ability to remain alert and select stimuli for extended periods, plays a key role in information processing, working memory, and other EFs [12,19]. This function is evident during the early stages of language acquisition, where children must focus on relevant linguistic input while filtering out irrelevant information. Sustained attention was significantly linked to sentence comprehension, grammatical skills, and narrative abilities in children with a DLD [15,20].

Research on sustained attention in the DLD population has revealed significantly poorer performance in measures of auditory sustained attention [21,22]. Nevertheless, the question was raised as to whether these differences primarily stem from auditory processing difficulties rather than sustained attention deficits [18]. Results regarding visual–spatial sustained attention in children with a DLD are mixed, with some studies indicating significant differences between groups, and others not [23,24]. Nevertheless, a meta-analysis conducted by Ebert and Kohnert [25] analyzed sustained attention studies in individuals with a DLD, including those employing variations in the Continuous Performance Task (CPT) behavioral test [26]. This widely used measure of sustained attention involves pressing a button upon presentation of a recurring target and withholding responses to non-target stimuli, either verbal or visual. In tasks of this nature, subjects with sustained attention deficits struggle to select target stimuli consistently over time, leading to missed targets, incorrect responses to distractors, or both. 

Ebert and Kohnert [25] revealed significant deficits in sustained attention in both auditory and visual–spatial modalities, where the DLD population exhibited low accuracy compared to their typically language developing (TLD) peers. Notably, this difference was not observed in response times. Subsequent studies have reported similar results in the pediatric population, employing the CPT in both verbal and nonverbal modalities [11,20,27].

While group comparisons between children with a DLD and their TLD peers highlight significant deficits in linguistic and cognitive domains, these group-level findings often fail to capture the considerable variability present within the DLD population [16,28]. Individual characteristics, such as the severity of language deficits, variability in executive functions (e.g., working memory and sustained attention), and the interplay between these domains, reveal a heterogeneous profile among children with a DLD. Kapa and Erikson [24] conducted an analysis of 11 scientific studies reporting performance in sustained attention, working memory, inhibition, and flexibility in individuals with a DLD and their TLD peers. While the mean comparisons indicate a general trend of subjects with a DLD scoring lower than their TLD counterparts, both groups displayed wide standard deviations, suggesting that some subjects with a DLD obtained scores similar to TLD children.

In light of the reported findings, it is clear that the cognitive deficits in children with a DLD are diverse, but there is consensus that a significant number of individuals experience these difficulties. According to cognitive models associated with executive function [3,4,5], deficits in perceptual processing, working memory, and attention are among the most prevalent in this population. To address these challenges, this research incorporates two nonverbal tasks designed to evaluate key cognitive mechanisms. The first, Duno and the Worms, assesses sustained attention through visuospatial tasks that require stimulus inhibition, while the second, Torpo the Clumsy Mole, measures working memory by evaluating the learning of spatial sequences mediated by perceptual tasks. These tests were specifically chosen because they integrate perceptual elements with representative cognitive functions and were validated for use with the Chilean population at this critical early stage of development.

The training and assessment of executive functions in children present significant challenges. A survey conducted in educational settings in the United States involving 2961 speech–language pathologists revealed that only half of the specialists reported implementing cognitive-level interventions for preschoolers [29].

Given this scenario, there is a growing interest in investigating how skills that have been shown to be impaired, such as sustained attention and working memory, influence language development. In this context, our aim is to compare the performance of sustained attention and working memory in preschool children with a DLD, native Spanish speakers, and their TLD peers; we hypothesize that the children with a DLD will demonstrate lower performance in both sustained attention and working memory compared to their TLD peers. This approach emphasizes the importance of cognitive-level interventions that complement traditional language-focused strategies, offering a more holistic framework that supports children with a DLD.

## 2. Materials and Methods

This quasi-experimental study entailed the use of two neuropsychological tests for the assessment of performance in working memory and sustained attention.

### 2.1. Participants

The group of participants in this study consisted of 30 children with a DLD in the experimental group and 30 TLD children in the control group. For the estimation of the minimum required sample size, the following parameters were taken into consideration: (a) effect size (f) = 0.25; (b) statistical power (1 − β) = 0.95; (c) significance level (α) = 0.05; and (d) number of measurements = 2. According to these variables, a minimum of 27 individuals per group is needed, as calculated by the G*Power program version 3.1.7. [30].

The inclusion criteria stipulated participants falling within the age range of 4 years to 4 years and 11 months during the assessment and actively attending preschool level. Additionally, it was required that their vision be either normal or corrected. Participants falling in the DLD group were students at a special language school where they are evaluated upon admission in accordance with Chilean ministerial regulations. This involves taking the Teprosif-R, Tecal, and STSG tests, which are used jointly for the diagnosis of DLDs [31]. The evaluation of the children was made by different speech–language pathologists.

Due to the impracticality of reproducing the evaluation instruments mandated by educational regulations within a 6-month period, we opted to employ the Test of Initial Language Development (TELD-3:S) by Ramos et al. [32]. This tool was used to validate the linguistic diagnosis of the participants. This test was selected because it was validated in Chile for diagnosing DLDs, with high reliability indicated by Cronbach’s Alpha coefficients of 0.931 in the receptive subtest, 0.947 in the expressive subtest, and 0.969 for the total test score. The application of the tool was overseen by a speech–language pathologist. The TELD-3:S is used to assess the ability to follow instructions through the use of concrete materials. In semantic terms, the test evaluates the identification of vocabulary through visual stimuli mixed with distractors. On the grammar level, it assesses sentence identification in a setting that also includes distractors.

On the expressive level, the test has participants name visually presented elements and respond to direct informational questions. These are crucial abilities evaluated for the diagnosis of DLDs [1]. The score for a subject to be included in the DLD group must be at or below the 10th percentile for their age. Conversely, subjects in the TLD group score above the 25th percentile. As reflected in Table 1, significant differences in TELD-3:S scores are found between the groups.

Regarding exclusion criteria, none of the individuals included in the sample exhibited any identified developmental or sensory disorders, such as cognitive impairments, autism, or cerebral palsy. Furthermore, none of the participants had a documented history of trauma necessitating medical intervention. Such information was collected during anamnesis responses provided by parents or guardians in an initial interview.

Written consent was obtained from parents and/or legal guardians, and participants also provided verbal consent before participating in the research. This study received ethical approval from the Ethics, Bioethics and Biosafety Committee (Protocol No. CEBB 731-2020) of the University of Concepción (Chile).

### 2.2. Instruments

All of the instruments described below were administered individually in a room with only the participant and the speech therapist present.

#### 2.2.1. Linguistic Assessment

The linguistic diagnosis of the participants was validated using the TELD-3:S, adapted by Ramos et al. [32]. This tool assesses the receptive and expressive language abilities of individuals aged 2 to 7 years and 11 months. The receptive subtest comprises 37 items (13 grammatical and 24 semantic) designed to assess a child’s comprehension of spoken language. Early items involve basic questions, such as *Show me the dog* or *Show me the shoe,* progressing in complexity to tasks such as *Show me the girl climbing the ladder.* Before the end of the test, participants are presented with questions like *Which word is unrelated to the others: bed-table-dad-chair?*

The expressive subtest comprises 18 grammatical and 21 semantic items, including tasks like repeating sentences and responding to inquiries such as *How old are you?* In the later stages of the test, participants engage with more complex questions like *Look at the shoe, coat, trousers and T-shirt. They are all...*

Using the outcomes from TELD-3:S, we categorized the participants into two study groups. Those with DLDs achieved scores at or below the 10th percentile, whereas TLD children obtained scores at or above the 25th percentile (refer to Table 1 for individual participant scores).

#### 2.2.2. Working Memory Assessment

In order to assess the working memory of participants, the nonverbal task “*Torpo el topo torpe/Torpo the clumsy mole*” from the Childhood Neuropsychological Assessment Test was used (original Spanish version: Test de Evaluación Neuropsicológica Infantil, TENI [33]). Originating and validated in Chile, this instrument gauges working memory, boasting a Cronbach’s reliability index of 0.8. This nonverbal task entails recalling a visual sequence and taps into the participant’s ability to temporarily hold and manipulate visual information in their working memory [34]. It was successfully used in Chile to measure working memory functioning in 5-year-old subjects [35].

Participants watch a mole emerging in various holes within a 3 × 3 grid on a tablet screen. The children are informed that the mole is lost and is probing the holes to find an exit. The mole materializes in two holes in sequence and then disappears. Following a bell sound, the child must replicate the order in which the mole appeared. The sequence progressively expands to eight positions. Practice trials are administered and reiterated until the child comprehends the instructions entirely before advancing to the test itself. The task concludes when the child fails two consecutive trials, and the analysis is founded on the accuracy of the responses.

#### 2.2.3. Sustained Attention Assessment

Sustained attention was assessed using the nonverbal task “*Duno y los gusanos/Duno and the worms*” from the TENI [33]. Originating and validated in Chile, this instrument gauges sustained attention, boasting a Cronbach’s reliability index of 0.8 This visuospatial task is based on the CPT, a widely used model of sustained attention developed by Haldor Rosvold in 1956 [25,26]. In TENI’s version, the child observes a conveyor belt of apples for 6 min and is required to touch the screen each time one of these apples has a worm, and to refrain from touching when the apple lacks the worm. The task is presented in a tablet screen format and includes practice trials before the test. Two measures of analysis are obtained, including response time (RT), which is the average time from the presentation of the target stimulus until the child touches the screen. This variable is calculated as the sum of the response times for all correct responses associated with the target stimuli divided by the number of correct responses. The second measure is the accuracy index, which is the number of correct responses divided by the number of errors. The test was successfully used to measure sustained attention functioning in 6-year-old subjects [36].

#### 2.2.4. Procedure and Statistical Analysis

A descriptive analysis was conducted to summarize the performance measures of Torpo the Clumsy Mole’s accuracy, Duno and the Worms’ reaction time (RT), and Duno and the Worms’ accuracy within each group (DLD and TDL). Additionally, an inferential analysis was performed, mediated by the results of the Kolmogorov–Smirnov test assessing sample normality and size (N = 60), to compare the means of these variables between the two groups. The outcome of the Kolmogorov–Smirnov test determined whether parametric or non-parametric tests were employed. Non-parametric tests, such as the Mann–Whitney U test, are more suitable when data violate the assumption of normality, especially when the sample size is small or the data are skewed. These tests provide more accurate results in such cases, as they do not rely on the assumption of a normal distribution that parametric tests require [37].

The statistical analyses were conducted using SPSS 22 [38].

## 3. Results

The Smirnov test results suggest that only sustained attention reaction times followed a normal distribution (*D*(60) = 0.107, *p* = 0.082). Both working memory and sustained attention accuracy variables exhibited significant deviations from normal distribution (*D*(60) = 0.183, *p* < 0.001 and *D*(60) = 0.171, *p* < 0.001, respectively). Due to the sample size and non-normal distribution of data, non-parametric tests were employed for the statistical analysis of working memory and sustained attention accuracy variables.

We first present the results of the Student’s *t*-test for the variable with normal distribution and then the Mann–Whitney U test for variables with non-normal distribution.

Regarding sustained attention reaction times, the results indicated that participants with a DLD achieved significantly shorter reaction times compared to the control group (*t*(58) = −6.15; *p* < 0.005), as is consistent with previous findings [25]. The effect size, as indicated by G*Power analysis, was *d* = 1.59, and the power was 1 − β = 0.99. In the accuracy index of sustained attention, the DLD subjects exhibited significantly lower performance than the TLD group (*U* = 96, *z* = −5.27, *p* < 0.005). The effect size, as indicated by G*Power analysis, was *d* = 1.88, and the power was 1 − β = 0.99 (see Table 2), aligning with previous research [11,20,22,27].

The analysis of working memory measures revealed a significantly lower average of correct responses in the “*Torpo the clumsy mole/Torpo el topo torpe*” task in the DLD group compared to their TLD peers (*U* = 142.5, *z* = −4.61, *p* < 0.005). The effect size, as indicated by G*Power analysis, was *d* = 1.47, and the power was 1 − β = 0.98. (see Table 2). This suggests that the DLD group retains short visual information sequences to a lesser extent than the control group, as is consistent with findings from previous studies [14,17].

To investigate potential correlations between the variables, Spearman’s rho correlation coefficient (ρ) was employed. Correlations were found among all three variables, as shown in Table 3. These findings suggest a significant linear relationship between working memory performance and sustained attention, both for accuracy and reaction times.

## 4. Discussion

This study investigates the performance during working memory and sustained attention tasks by Spanish-speaking preschool children with a DLD compared to their TLD peers. Our results reveal significant deficits in both working memory and sustained attention in DLD participants, providing valuable insight into the multifaceted nature of DLD during the early school years. Originally conceptualized as Specific Language Impairment (SLI), the evolving definition of DLD now encompasses not only linguistic challenges but also broader cognitive dimensions [2].

In line with previous research [14,15], our results support the existence of working memory deficits in children with a DLD. The compromised ability to retain and manipulate short sequences of visual information distinguishes the DLD group from their TLD counterparts. Comparable evidence in different languages supports our findings: Ralli et al. [39] found limited working memory capacity in 8- to 9-year-old Greek-speaking children; Marini et al. [40] reported that measures of executive function, particularly updating working memory and inhibition, correlated with linguistic and narrative measures in Italian preschoolers with a DLD; and Acosta et al. [41] found working memory deficits in Spanish-speaking children aged 5–11 years with a DLD. In addition, a longitudinal study by Blom and Boerma [42] found severe and persistent visuospatial working memory deficits in 5-year-old children with a DLD. Gillam et al.’s [43] results further support the persistence of these deficits by demonstrating differences in children between the ages of 7 and 11.

Our research is consistent with the findings of working memory deficits in the DLD population and provides new evidence within a Spanish-speaking population under the age of 5. In a previous study conducted in Spanish [35] with children aged 5 to 6 years, a task utilizing an experimental paradigm revealed higher accuracy scores for regular verbs compared to irregular verbs, across both present and past tenses. These findings were further influenced by visual working memory, with the DLD group showing modulated reaction times. This measure was assessed using the same TENI task utilized in our research (Torpo the Clumsy Mole). Collectively, these experiments underscore the significance of working memory in early developmental processes, particularly in children with a DLD.

In terms of sustained attention, we observed a distinct pattern in the children with a DLD compared to their TLD counterparts, characterized by shorter reaction times. This reverse situation is consistent with previous studies that also reported an absence of reaction time deficits in the DLD group [25]. A possible explanation for this discrepancy could be that the TLD group took more time to visualize and confirm the target before pressing, whereas the DLD group anticipated their response, resulting in faster reaction times but a higher proportion of errors, as reflected in their accuracy performance. This is consistent with previous studies showing deficits in sustained attention in individuals with a DLD [18,23,25]

Our results, which to our knowledge are the first to study sustained attention performance in individuals with a DLD under the age of 5, provide important clues for the assessment and diagnosis of this population. By using a dual-task experimental paradigm, a previous study conducted with Spanish-speaking children aged 6 to 7 years [36] found that the sustained attention covariate modulated linguistic outcomes associated with reading sentences in the past and future tenses. This implies that the introduction of the attention covariate led to interactions with verbal tense and direction of movement in the representation of a conceptual metaphor, especially in children with a DLD. These results demonstrate the developmental nature of sustained attention and highlight the fact that the effect of attention can be observed at such an early age, in accordance with the results of the present investigation.

The importance of determining the performance of these two variables lies in the consistent relationship between working memory and language development in DLDs. Recent research also links sustained attention to morphosyntactic skills, vocabulary comprehension, and picture naming latency [20,21,44]. In addition, sustained attention was directly related to working memory performance [18] or, according to the joint mechanism deficit hypothesis, as a possible cause of working memory limitations [45].

The correlation analysis conducted in our study revealed significant relationships among the variables of working memory performance and sustained attention accuracy for both the DLD and TLD groups. Specifically, we found a positive correlation between working memory performance and sustained attention accuracy in both groups, indicating that individuals with better working memory abilities tend to exhibit higher levels of sustained attention accuracy. This finding suggests that the ability to retain and manipulate information in working memory may contribute to the sustained attention required for tasks such as Torpo the Clumsy Mole. Additionally, a positive correlation was observed between working memory performance and sustained attention accuracy in both groups, suggesting that individuals with better working memory abilities tend to respond more quickly during sustained attention tasks. These correlations highlight the interconnected nature of cognitive processes such as working memory and sustained attention and underscore their collective influence on cognitive functioning in individuals with language disorders.

It is important to note that although significant differences were found between the two groups, the data did not represent ranges of normality due to individual differences in some subjects. This highlights the inherent heterogeneity in cognitive function profiles between DLD and TLD children and underscores the need for personalized interventions that go beyond a one-size-fits-all approach and recognize and address the unique cognitive needs of each child. In particular, Kapa and Erikson [24] have emphasized the importance of tailoring interventions to individual strengths and weaknesses in executive functioning. In addition, findings from Smolak et al. [18] and Zapparrata et al. [5] support the notion that a personalized approach may lead to more effective outcomes given the variability in cognitive profiles within the DLD population.

Previous research has demonstrated improvements in executive functions, such as working memory and sustained attention, through computerized cognitive training in clinical settings [46,47] and educational environments [48]. Game-based training has shown promise in improving outcomes for both DLD populations [49] and typically developing children [50].

### Limitations

A limitation of our study is the absence of nonverbal IQ measurement in both DLD subjects and TLD peers. However, the use of this variable in our study can be controversial due to several reasons. Firstly, nonverbal IQ has traditionally been primarily utilized to equalize performance between DLD and TDL groups; however, these comparisons have been conducted in studies with populations older than those in our investigation [38,39,51]. Moreover, there is no validated test available in our country to measure IQ in 4-year-old subjects, with the recommended Wechsler Intelligence Scale for Children: Fifth Edition typically administered from the age of 6 [52]. Therefore, the absence of a validated test for younger subjects poses an additional challenge in accurately measuring nonverbal IQ. Secondly, there is no consensus on the most appropriate method for considering this variable. Other approaches, such as that of Larson and Weister [14], have presented significant differences in nonverbal IQs between DLD and TDL groups, potentially categorizing DLDs more as a cognitive disorder than a language impairment, which can be misleading. Finally, our focus was on language assessment, and we considered that our subjects underwent a rigorous evaluation using instruments that assure linguistic diagnosis and their capacity to respond to tests. There were no indicators of IQ differences, which was also noted in the interviews with caregivers.

Moving forward, future research should address these methodological challenges and strive to adopt standardized approaches to control for nonverbal IQ while ensuring an accurate depiction of the unique characteristics of DLDs. Additionally, we suggest the use of specific linguistic tasks tailored to different language proficiency levels and the conduct of replications across different school levels to provide further insight into the trajectory of cognitive skills and language acquisition in the Spanish language context.

## 5. Conclusions

In conclusion, our study contributes significantly to the knowledge of the role of cognitive functions, especially working memory and sustained attention, in DLDs. It highlights that difficulties in these functions are present at an early preschool stage in Spanish-speaking children. The need for personalized interventions tailored to the unique cognitive profiles of children with a DLD, even before the preschool stage, is apparent. Stimulating cognitive domains not only benefits the clinical population, but also extends the positive effects to TLD peers. The potential use of game-based intervention in both populations highlights a promising avenue for future research.

The need to integrate interventions for cognitive functions, particularly working memory and sustained attention, with traditional language interventions becomes apparent, particularly in light of the gap reported by speech–language pathologists in incorporating cognitive-level interventions in educational settings [29]. Our study supports the argument for a holistic and integrated approach that addresses both linguistic and cognitive aspects to support preschool children with a DLD more effectively [53].

## Figures and Tables

**Table 1:** Groups’ TELD-3-S scores.

Variable	DLD (n = 30)			TD (n = 30)			Comparison	
	M	SD	Min–Max	M	SD	Min–Max		
Age (months)	55.03	3.85	48–60	56.17	2.52	49–60	*t* = −1.35	*p* = 0.18
TELD3-S rec	82.06	5.36	68–91	100.8	5.59	91–112	*t* = −13.24	*p* ≤ 0.001
TELD3-S exp	82.47	5.85	67–93	96.3	5.97	82–109	*t* = −9.06	*p* ≤ 0.001
TELD3-S total	164.53	3.48	156–169	197.1	5.42	185–204	*t* = −27.682	*p* ≤ 0.001

Note: Group mean values (M), standard deviation (SD), and score from TELD3-S.

**Table 2:** Means and standard deviations of working memory and sustained attention test for DLD and TLD participants.

	DLD (n = 30)			TLD (n = 30)		
Dependent Variable	M	SD	Min–Max	M	SD	Min–Max
Accuracy: Torpo the Clumsy Mole *	6.03	1.75	4–10	9.33	2.64	5–13
Reaction time: Duno and the Worms *	6.8	2.37	3–15	10.6	2.42	5–15
Accuracy index: Duno and the Worms *	7.167	1.88	5–12	11.07	2.26	6–14

* = Indicates significant differences.

**Table 3:** Spearman’s rho correlations between executive functions.

	DLD			TLD		
Variable	ACC WM	ACC SA	RT SA	ACC WM	ACC SA	RT SA
ACC WM	1	564 **	0.624 **	1	0.463 **	0.603 **
ACC SA		1	0.630 **		1	0.463 *
RT SA			1			1

Note: ACC WM = accuracy working memory (Torpo the Clumsy Mole); ACC SA = accuracy sustained attention (Duno and the Worms); RT SA = reaction time sustained attention (Duno and the Worms RT). * = Indicates significant differences at 0.05, ** = indicates significant differences at 0.01.

## Data Availability

The data generated and analyzed in this study are available on reasonable request from the corresponding author. The data are not publicly available as they are human data from adults and children in neurotypical and clinical groups.
